# The effectiveness of health‐related quality of life (HRQOL) model in people with HIV: A cross sectional study

**DOI:** 10.1002/hsr2.2217

**Published:** 2024-07-12

**Authors:** Mahsa Abdollapour, Seyed Ahmad Seyed Alinaghi, Amir Sam Kianimoghadam, Abbas Masjedi‐Arani, Maryam Bakhtiari, Seyed Ali Dehghan Manshadi, Mohsen Rostami, Minoo Mohraz

**Affiliations:** ^1^ Department of Clinical Psychology, School of Medicine Shahid Beheshti University of Medical Sciences Tehran Iran; ^2^ Iranian Institute for Reduction of High‐Risk Behaviors Tehran University of Medical Sciences Tehran Iran; ^3^ Department of Clinical Psychology, Taleghani Hospital Research Development Unit, School of Medicine Shahid Beheshti University of Medical Sciences Tehran Iran; ^4^ Department of Infectious Diseases and Tropical Medicine, Imam‐Khomeini Hospital Complex Tehran University of Medical Sciences Tehran Iran; ^5^ National Defense University of Tehran Tehran Iran

**Keywords:** Health‐related quality of life (HRQOL), HIV, mindfulness, sleep

## Abstract

**Background and Aims:**

Acquired immune deficiency syndrome (AIDS) are a chronic and disabling disease that has a significant impact on quality of life due to weakening of physical health, financial problems and social stigma. This study aims to validate the Health‐related quality of life (HRQOL) model in people with human immunodeficiency viruses (HIV) in Iran.

**Methods:**

Four hundred and fifty‐two people with HIV from Imam Khomeini Hospital between the age of 18 and 65 years (men: 308, women: 144) participated in the research. anonymously completed a battery of questionnaires, namely the Persian basic psychological need satisfaction and frustration scale, SF‐36, PSQI and mindful attention awareness scale. The method of the present study was the structural equation model.

**Results:**

Current findings indicated there is a significant positive between mindfulness and need satisfaction, physical and mental health and, significant negative between mindfulness and quality and quantity of sleep. Mindfulness and need satisfaction are significant positive predictors of quality of life in people with HIV. Quality and quantity of sleep are significant negative predictors of quality of life in people with HIV. There is a positive significance between need satisfaction and physical and mental health but there is a negative significance between need satisfaction and quality and quantity. there is a negative significant between the quality and quantity of sleep with physical and mental health. Mindfulness facilitates the satisfaction of more psychological needs and improves the quality of sleep. The quality of sleep is a negative significant predictor for physical and mental health but the quantity of sleep is a negative significant predictor for physical health.

**Conclusion:**

The HRQOL model can explain 18% of physical health and 16% of mental health in people with HIV in Iran. The elements of this model can be useful in evaluating and treating people with HIV in the Iranian Population. They also can use the models to plan for better services.

## 1 INTRODUCTION

The human immunodeficiency viruses (HIV) has established itself as a persistent and controllable affliction, no longer an immediate death sentence.^1^ This insidious infection is responsible for countless cases of ill health and loss of life across continents, particularly in sub‐Saharan Africa. Though efforts have been exerted to combat this grave dilemma, HIV remains an enduring presence among the most widespread and incurable infectious diseases known to man.^2^ Citing statistics from the World Health Organization (WHO), it becomes evident that approximately 75 million people worldwide have fallen victim to HIV over time, resulting in a tragic toll of approximately 32 million lives lost since the onset of its devastating epidemic despite the significant progress made in combating HIV, it is disheartening to note that a staggering 37.9 million individuals worldwide still grapple with this debilitating virus as of 2018.^3^, ^4^ The WHO emphasizes that HIV remains one of the foremost causes of death on a global scale.^5^ In examining the prevalence of this pandemic at a global, regional, and national level, it becomes evident that Africa bears the highest burden in terms of HIV cases.^6^ Despite an overall decline in new infections across the globe, certain countries like Iran are witnessing an alarming increase in HIV incidence rates. According to estimates derived from the United Nations Program on HIV/AIDS (UNAIDS) spectrum and modeling techniques for the year 2019, approximately 59,000 and 64,000 in 2022 people currently reside with HIV within Iran's borders.^4^ Furthermore, each year brings about around 4100 fresh instances of infection along with roughly 2500 AIDS‐related fatalities within the country. it is crucial to acknowledge that acquired immune deficiency syndrome (AIDS) represents more than just a physical ailment; rather it permeates into all aspects of an individual's life. Beyond its impact on their health status alone, those afflicted by this chronic infection experience profound effects on their social relationships and mental well‐being.^7^ The diagnosis of HIV/AIDS significantly impacts the overall well‐being and psychological state of individuals. Their physical health status and their quality of life (QOL) are crucial factors in assessing their ability to manage a chronic illness or condition.^8^ The concept of “quality of life” (or QOL) can be traced back to discussions about health definitions and an individual's functional capacity.^9^ Yet, today the WHO defines QOL as one's assessment and evaluation of their present existence, taking into account community values, cultural norms, as well as aspirations, objectives, and worries The concept of QOL encompasses various facets in the lives of individuals afflicted with chronic illnesses.^10^ Fluctuations in Health‐Related QOL (HRQOL) encompass alterations in functional capabilities, perceptions from others, social opportunities, treatment requirements, and disability levels; all aspects that may persist throughout an individual's lifetime we draw upon the profound principles of self‐determination theory (SDT), an extensive framework exploring human motivation and the factors that either foster or hinder human growth.^11^ SDT offers a valuable lens through which to examine the predictors of HRQOL, as it outlines the essential psychological needs for autonomy, competence, and relatedness that are inherent in all individuals seeking optimal functioning.^11^

Autonomy encompasses a deep sense of volition and wholehearted endorsement in one's actions; competence involves feeling capable and effective in accomplishing desired outcomes; while relatedness entails experiencing reciprocal care and intimacy with others.^12^ The connection between these intrinsic needs and overall well‐being is thoroughly substantiated by numerous studies revealing that their fulfillment positively correlates with various measurements of happiness such as life satisfaction, while the deprivation of these needs is negatively associated with malaise including depression and anxiety.^13^ There exists certain evidence indicating that these inherent psychological needs bear influence in delineating the HRQOL. While it is firmly established that attaining satisfaction with such psychological needs correlates strongly with overall well‐being, the underlying mechanisms fostering this association are yet to be explored.^14^

An additional influential component in the mental well‐being of individuals affected by AIDS is the quantity and quality of their sleep. As per an extensive meta‐analysis, approximately 58% of those with HIV endure challenges related to their sleep patterns.^15^ Those afflicted with insomnia are prone to experiencing numerous unsettling symptoms throughout their waking hours, which can have profound repercussions across various aspects of life, including social interactions, familial bonds, and interpersonal relationships eventually compromising these individuals' overall quality of life. Sleep disruption gives rise to a myriad of psychological issues such as despondency, anxiety, and impaired cognitive faculties. In light of preliminary evidence suggesting that fulfilling one's psychological needs may yield advantageous effects on people with HIV, sleep patterns and overall well‐being, it becomes imperative to ascertain the factors contributing to enhancing this sense of gratification.^16^, ^17^

It is worth noting that sleep disruptions are prevalent within people with HIV, estimated to affect as many as 70% according to several studies conducted during the early stages of the HIV epidemic within the HIV population, there exists an overpowering anxiety surrounding the possibility of succumbing to a fatal illness.^18^ The burdensome weight of financial concerns, coupled with the harsh reality of stigmatization and episodes of depression, exacerbate this pre‐existing anxiety. Furthermore, unemployment looms as a persistent issue within this community.^19^ These anxieties can inflict grave disruptions upon physical, mental, and emotional well‐being and subsequently impact the tranquility of sleep experienced by individuals. These disturbances in sleep quality not only impair one's overall vitality but also hinder cognitive function a hindrance that may result in inadequate adherence to prescribed medications.^18^ Previous research has convincingly demonstrated a strong correlation between fulfilling basic psychological needs (BPN) and enjoying an elevated quality of life. Consequently, it becomes necessary to delve deeper into exploring the intricate nature of binding BPNs with emotions such as depression and their subsequent effect on the overall quality of life experienced by people with HIV.^20^ This investigation holds particular importance when considering that those infected often face additional psychosocial adversities; they may suffer from a lack of social support or grapple with unconquerable stigma while simultaneously lacking proficient coping mechanisms all factors which enable these challenges to significantly compromise their individual qualities of life.^21^

One of the conceivable factors contributing to the fulfillment of our needs, an area that has garnered significant attention in scholarly mental health investigations, is the principle of mindfulness. Mindfulness involves intentionally redirecting one's focus to the present moment without forming judgments, a skill that can be honed through meditation or other forms of training.^22^ Through the development of greater satisfaction with our fundamental psychological necessities, we create a receptive mental environment wherein mindfulness can thrive and subsequently enhance our overall well‐being. Numerous studies examining correlations and interventions rooted in mindfulness have revealed its positive impact on sleep quality and duration. By adopting a conscious and accepting mindset towards the arousal processes hindering sleep, mindfulness allows individuals to attain a more peaceful night's rest.^23^

The evaluation of the HRQOL model has been done among target groups and different cultures, but it has not been done in Iran and among the group of people with HIV, so the aim of this study is the effectiveness of HRQOL model in people with HIV. We specifically examined five hypotheses: 1. There is a relationship between meeting needs and physical and mental health in people with HIV disease. 2. There is a relationship between the satisfaction of needs and the quantity and quality of sleep in people with HIV disease. 3. There is a relationship between the quantity and quality of sleep with physical and mental health in people with HIV disease. 4. There is a relationship between mindfulness and the quantity and quality of sleep in people with HIV. 5. There is a relationship between mindfulness and satisfaction of needs in people with HIV disease.

## 2 METHODS

### 2.1 Participants

Participants in the present study were initially estimated to be 452 individuals using Free Statistics Calculators version 4.0 software. Therefore, considering a significance level of 0.05, power of 0.90, the effect size of 0.19, 89 visible variables, and 6 hidden variables, the sample size software estimated 450 samples.^24^ However, to compensate for the additional 10% missing data, 495 samples were collected instead of the estimated sample size, and after preprocessing, 452 samples remained for the final analysis. The sample collection method was convenience sampling. The sampling location was the counseling center of Imam Khomeini Hospital and Ahmadi counseling center in Tehran. The time of data collection was 4 months (October 29, 2019–March 14, 2020). Participants in the study included single and married individuals, both male and female, aged 18–65 years (308 males and 144 females). The inclusion criteria for this research include HIV diagnosis by an infectious disease specialist, the ability to read and write in Persian, refer to counseling centers for behavioral diseases, desire to participate in research, and being between 18 and 65 years old and exclusion criteria inclusion Disruption of collaboration between the patient and the behavioral disease counseling center, fatigue of the patients, failure to complete the questionnaires.

### 2.2 Measure

#### 2.2.1 The basic psychological need satisfaction and frustration scale (BPNSFS)

The basic psychological need satisfaction and frustration scale was designed by B Chen. This scale includes 24‐item and 6 subscales including autonomy satisfaction, relatedness satisfaction, competence satisfaction, autonomy frustration, relatedness frustration, and competence frustration. Four items were used to assess each factor, using a 5‐point Likert scale ranging from 1 (not at all true) to 5 (very true). The internal consistency of BPNSFS in the original version using Cronbach's alpha that for satisfaction needs was 0.73–0.89 and for frustration needs was 0.64–0.86.^25^ Also, in the research of Nishimura and Suzuki, the internal consistency for the satisfaction components of, autonomy, relatedness and competence were 0.77, 0.74, and 0.72, respectively, and for the frustration components of, autonomy, relatedness and competence were 0.75, 0.78, and 0.71. In addition, the internal consistency of the total component of satisfaction of needs was calculated as 0.82 and frustration of needs as 0.83. To examine convergent validity, The correlation between the components of satisfaction of needs with mental vitality for autonomy satisfaction, relatedness and competence were 0.19, 0.23, and 0.42 and the correlation between the components of frustration needs with depression, for the frustration of autonomy, relatedness and competence is 0.26, 0.35, and 0.17 were obtained.^26^ Moreover, the psychometric properties of the Persian version of the BPNSFS were evaluated among a sample of university students in Iran. The results revealed that the Persian BPNSFS has appropriate internal consistency(*r* = 0.84).^27^

#### 2.2.2 Health survey short form (SF‐36)

The SF‐36 was designed by Ware and Sherbourn for use in clinical practice and research, health policy evaluation, and general population surveys. SF‐36 was a generic HRQOL instrument that measures eight different dimensions of life which are calculated as 8 domains: physical functioning (PF), role physical (RP), bodily pain (BP), general health (GH), vitality (VT), social functioning (SF), role emotional (RE), and emotional well‐being (EW). Scoring and calculation of scales were performed by using Ware's survey manual based on these eight domains, two summary composite scores were constructed: the Physical Health Composite score (PHC) and the Mental Health Composite score (MHC). Finally, the Total quality of life score (TQL) is calculated. PHC, MHC and TQL were used for further analyses in this study. Higher values of all SF‐36 scores mean better functioning and well‐being.^28^ SF‐36 was appropriate test‐retest reliability for all eight domains (*r* = 0.75–0.92). Internal consistency was also acceptable for all domains (Cronbach's alpha = 0.73–0.90). content validity was found to be good.^29^ The results revealed that the Persian version of SF‐36 has appropriate Internal consistency by using Cronbach's alpha (0.91). the convergent validity for each item ranged from 0.57 to 0.69 for physical functioning (PF) scales, 0.61–0.70 for role physical (RP) scale, 0.85–0.90 for bodily pain (BP) scales, 0.64–0.74 for general health (GH) scales, 0.62–0.75 for vitality (VT) scales, 0.77–0.88 for social functioning (SF) scales, 0.56–0.73 for role emotional (RE) scales and 0.69–0.77 for mental health (MH) scales.^30^

#### 2.2.3 Pittsburgh sleep quality index (PSQI)

PSQI was designed by D.J. Buysse to examine the measures of sleep quality and disorder retrospectively over 1 month using self‐reports. The PSQI contains 19 items that produce a universal sleep quality score and the following seven component scores: sleep quality, sleep latency, sleep duration, habitual sleep efficiency, sleep disturbance, use of sleeping medications, and daytime dysfunction, and is graded on a 4‐point Likert scale from “0” to “3”. The seven component scores are then added to yield one “global” score, with a range of 0–21 points, “0” indicating no difficulty and “21” indicating severe difficulties in all areas results showed a good internal consistency using Cronbach's alpha (*r* = 0.83).^31^ The results revealed that the Persian version of the PSQI has appropriate internal consistency (*r* = 0.89) and good validity (0.86).^32^

#### 2.2.4 Mindful attention awareness scale (MAAS)

MAAS was designed by Brown, K. W., & Ryan and has been usually used to assess for evaluate mindfulness. The MAAS is a 15‐item, 6‐point Likert‐type scale (6 = rarely; 1 = almost always) designed. The internal consistency of this questionnaire was obtained using Cronbach's alpha (*r* = 0.80) Also, this scale has demonstrated appropriate validity by using confirmatory factor analysis.^33^ Moreover, the psychometric properties of the Persian version of the MAAS were evaluated among a sample of university students in Iran. These results demonstrated acceptable levels of discriminant validity. The MAAS was evaluated using confirmatory factor analysis (CFA) and Rasch analysis. Results of the CFA showed a single‐factor structure with an acceptable fit to the data.^34^

#### 2.2.5 Procedure

The current study was approved by the Ethics Committee of Shahid Beheshti University of Medical Sciences. The type of present study is a cross‐sectional study and the statistical method is structural equation modeling. All participants received an HIV diagnosis from an infectious disease specialist during an examination. The objective and process of the research were explained to the participants, and with their consent, a written consent form was obtained assuring them of the confidentiality of their information. After receiving the research ethics code from the research ethics committee and obtaining informed consent from the participants, those who were literate in Persian and aged between 18 and 65 remained in the study. The current research sample initially consisted of 516 AIDS patients, but ultimately, due to patient non‐cooperation and pre‐processing, 452 individuals remained in the study. For statistical analysis of data, confirmatory and exploratory factor analysis has been used. The correlation matrix between the variables was used to examine and analyze the data, and the structural model of the research was constructed using linear structural relations (Lisrel) software. Azon boot was used to examine mediating effects. R and SPSS software were used for data analysis results.

## 3 RESULT

The present research includes 452 people with HIV, consisting of 308 men (68.1%) and 144 women (31.9%). The average the age and standard deviation of age for men are 33.63 and 7.89, respectively (Table 1).

**Table 1:** Sociodemographic characteristics of participants.

Variable	n	%
Gender	308	68.1
Male	308	68.1
Female	144	31.9
Marital status		
Single	221	48.9
Married	231	51.1
Highest educational level		
Secondary or less	216	47.8
Diploma	181	40.0
Bachelor	55	12.2
Occupation		
Employed	302	66.8
Unemployed	150	33.2

The average age and standard deviation of age for women are 33.06 and 8.15, respectively. The descriptive statistics of the study variables and the correlation between variables are reported (Table 2).

**Table 2:** Mean, standard deviation, and correlation for study variables.

Variable	M	SD			Correlations								
			1		2		3		4		5		6
1. Mindfulness	60.38	13.35	1										
2. Basic psychological need	40.09	10.94	0.343	**	1								
3. Sleep quality	7.82	3.54	−0.215	**	−0.299	**	1						
4. Amount of sleep	1.62	0.75	−0.258	**	−0.256	**	0.616	**	1				
5. Physical health	50.24	5.62	0.294	**	0.252	**	−0.372	**	−0.292	**	1		
6. Mental health	45.52	9.91	0.163	**	0.393	**	0.265	**	−0.182	**	0.108	**	1

A hypothetical model was created with the LISREL‐8 software output. Good fit indices for the hypothesized model are shown in Table 2. The conceptual model and correlation matrix between the latent variables of the research are seen in Table 3. As the contents of this table show, all correlations are appropriate (0.372 ≥ r ≥ 0.616). The results of Table 2 also show that mindfulness has a positive and significant relationship with need satisfaction, physical health and mental health. While it has a negative and significant relationship with sleep quality and sleep quantity. On the other hand, need satisfaction and physical and mental health have a positive and significant relationship with each other, while they have a negative and significant relationship with sleep quality and sleep quantity. Also, sleep quality and quantity have a negative and significant relationship with physical and mental health.

**Table 3:** Fit indices of the structural model of the research.

Fitness index	χ2	Df	χ2/df	NFI	CFI	IFI	GFI	RMSEA	SRMR
Calculated values	1.12	1	1.12	0.97	0.99	0.99	0.99	0.016	0.008
Accepted values	‐	‐	<5	>0.90	>0.90	>0.90	>0.90	<0.08	<0.08

The structural (conceptual) model of the research was tested using the LISREL software. After setting the structural equations, the aforementioned model was examined using the maximum likelihood method and the model fit was examined at two levels. At the first level, the overall fit of the model was examined based on fit indices. At the second level, the fit of the structural research model was examined based on the significance of path coefficients (structural coefficients). At the first level, after analysis, the overall fit of the conceptual model was examined. The fit indices of the conceptual model of the research reported in Table 3 indicate a very good fit of the model. All indices indicate a desirable fit of the conceptual model; Therefore, the structural model examining the mediating role of need satisfaction, sleep quality and sleep quantity in the relationship between mindfulness and physical and mental health has a good fit with the empirical data. At the second level, after initial analysis, the path coefficients of the model and coefficients of determination of the endogenous variables were examined.

**Figure 1:**
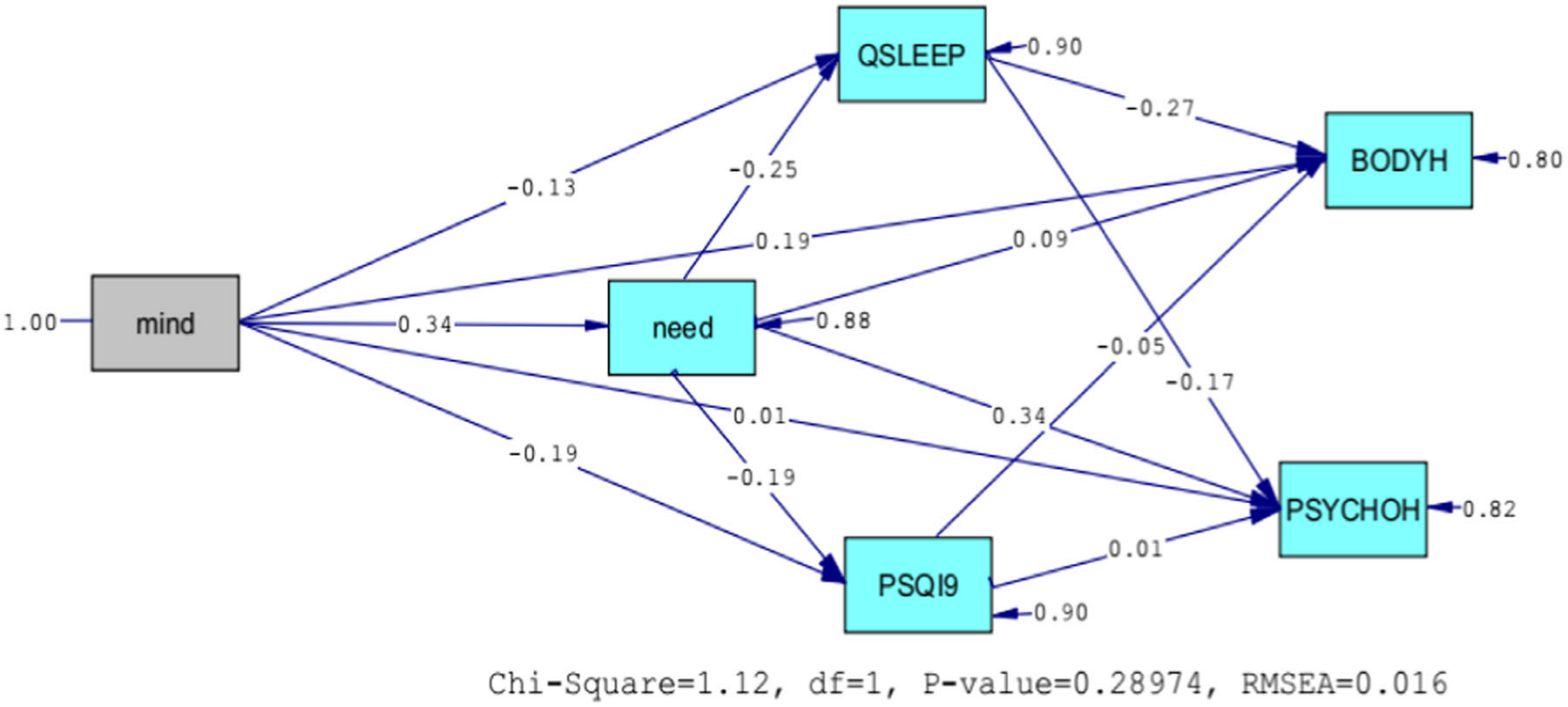
The initial structural model with the standardized coefficients.

Figure 1 shows the conceptual structural model along with the standardized coefficients.

**Figure 2:**
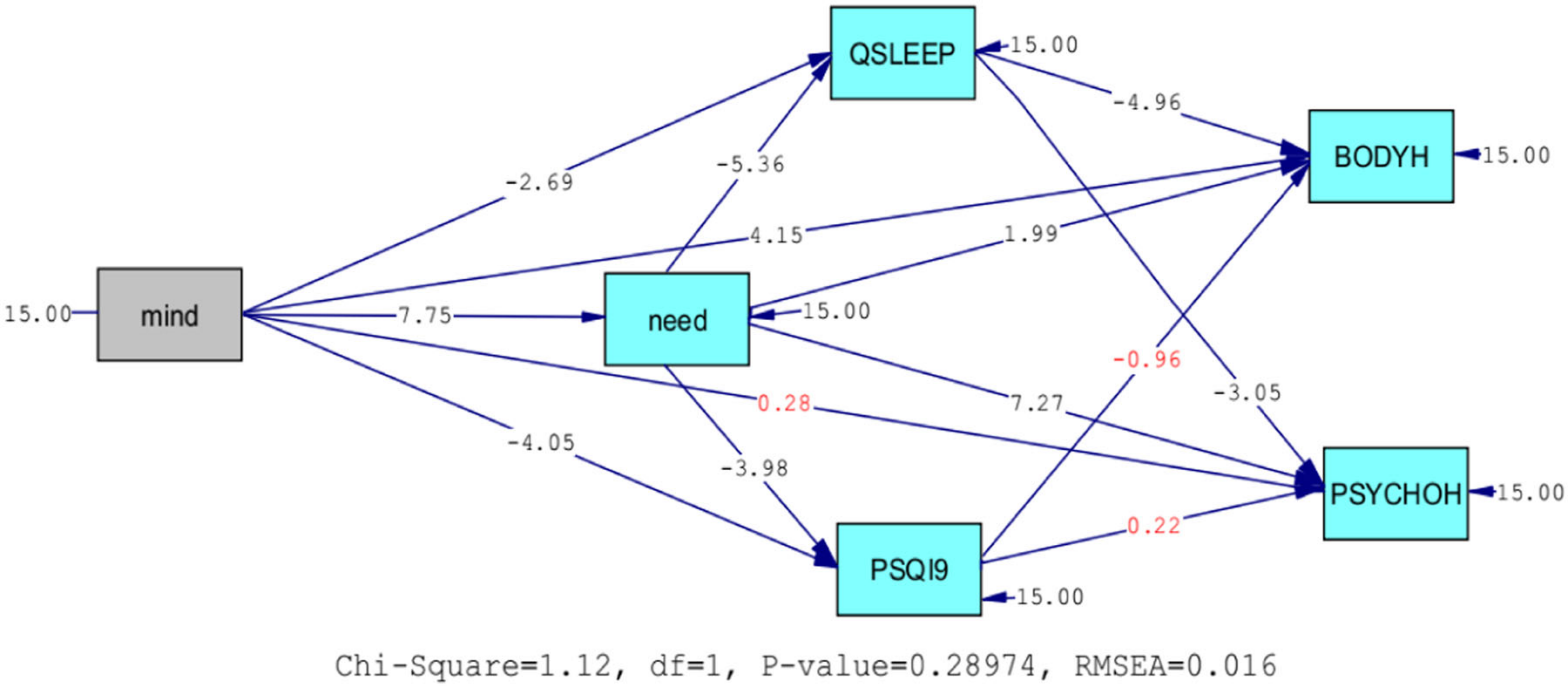
The initial structural model with the critical values.

The results presented in Figures 2 and 1 show that the path from mindfulness to mental health is not significant (*t* = 0.28, *p* > 0.01). Also, the path from sleep quantity to physical and mental health is not significant (*t* = −0.96, *p* < 0.001) and (*t* = 0.22, *p* > 0.01). On the other hand, the rest of the paths have significant coefficients. The coefficients of determination of the endogenous variables of the model are also moderate to low. In general, these results show that the present model can explain 18 percent of physical health and 16 percent of mental health. It also shows that mindfulness and need satisfaction can explain 10 percent of sleep quality and 9 percent of sleep quantity.

According to Table 4, the way of mindfulness of mind to physical health and mental health with the mediation of sleep quality is significant with standard coefficients of 0.035 and 0.022, separately, at *p* > 0.01 level. mindfulness mediating the amount of sleep with standard coefficients of 0.009 and 0.001 at *p* > 0.01 level has no significant effect on physical and mental health. furthermore, mindfulness through the mediates of the need for satisfaction sleep quality affects both physical health and mental health with standardized coefficients of 0.022 and 0.014 respectively at *p* > 0.01 level. While mindfulness mediating need satisfaction quantity of sleep on both physical health and mental health has no significant effect on physical health and mental health at *p* > 0.01 level.

**Table 4:** The results of the bootstrap test for mediating effects.

Independent variable	Mediation variable	Dependent variable		Standard coefficient	Standard error	Low range 0.95	High range 0.95	Significance level
Mindfulness	Quality of sleep	physical health		0.035	0.015	0.010	0.060	0.022
Mindfulness	Quantity of sleep	physical health		0.009	0.013	−0.012	0.032	0.444
Mindfulness	Quality of sleep	Mental health		0.022	0.012	0.003	0.040	0.049
Mindfulness	Quantity of sleep	Mental health		0.001	0.012	−0.022	0.017	0.843
Mindfulness	Need satisfaction	Quality of sleep	Physical health	0.022	0.007	0.012	ا0.036	0.001
Mindfulness	Need satisfaction	Quantity of sleep	Physical health	0.003	0.005	−0.004	0.011	0.466
Mindfulness	Need satisfaction	Quality of sleep	Mental health	0.014	0.006	0.005	0.025	0.017
Mindfulness	Need satisfaction	Quantity of sleep	Mental health	0.001	0.004	−0.008	ا0.006	0.851

## 4 DISCUSSION

The present study was conducted with the aim of the effectiveness of the Health‐related quality of life (HRQOL) model in Iranian people with HIV, which, in general, can explain 18% of physical health and 16% of mental health. The findings showed that mindfulness and need satisfaction are positive and significant predictors of health‐related quality of life (physical health and mental health).^20^, ^35^ Also, the results indicate that sleep quality and sleep quantity are negative and significant predictors of this type of quality of life.

The findings of the present study are consistent with previous studies.^36^, ^37^ The results of the present study showed that mindfulness has a positive and significant relationship with need satisfaction, physical health, and mental health. While it has a negative and significant relationship with sleep quality and sleep quantity. Instead, satisfaction of needs physical health and mental health have a positive and significant relationship, while sleep quality and quantity have a negative and significant relationship. Also Based on the findings of the present study, sleep quality has a negative and significant relationship with physical and mental health but sleep quantity has only a negative and significant relationship with physical health. Excessive sleep can lead to increased physical problems such as decreased physical activity, reduced energy, and increased heart disease. In individuals with AIDS, an increase in sleep hours is associated with more noticeable reductions in energy and physical activity, which are symptoms of the disease.^38^

In addition, in this study, the role of mindfulness in helping to satisfy basic psychological needs was investigated. mindfulness leads to optimum self‐regulation, permitting individuals to be in touch with their needs, values and emotions^39^, ^40^ As such, mindfulness is assumed to lead to more realistic evaluations (and, eventually, better satisfaction) of the three basic psychological needs for: competence (i.e. feeling effective in one's relations with the environment), autonomy (i.e. feeling choice, decision and willingness in one's actions) and relatedness (i.e. feeling linked with and loved by others).^41^ An increasing amount of research has reinforced this proposition.^42^, ^43^, ^44^

The findings confirmed our hypotheses. people living with HIV (PLHIV) who experienced more need satisfaction there is also a positive relationship with HRQOL and a negative relationship with poor sleep quality. This finding that satisfaction of needs leads to more physical and psychological health is in link with previous research in nonclinical populations, which showed that satisfaction of needs has a significant relationship with reducing symptoms of depression,^26^ and anxiety^45^ and increasing satisfaction^25^ with life and self‐esteem of adolescents.^46^

Poor sleep quality is associated with higher rates of depressive symptoms and physical problems in healthy individuals^47^ and sleep disorders are common in mood disorders (such as major depression) and cognitive disorders.^48^ In addition, sleep can have a two‐way relationship with health. Sleep disorders are not only related to blood pressure, diabetes and obesity.^49^ It has also been reported with symptoms of depression, physical illness and fatigue as factors related to low quality of sleep and short sleep duration.^38^ This study is by previous studies that have shown that sleep quality, not quantity, is related to physical‐psychological measures.

The relationship between physical and mental health has been proven in numerous studies that show that need satisfaction has a positive relationship with physical and mental well‐being (e.g., life satisfaction) and a negative relationship with psychological problems such as anxiety and depression.^50^ According to the theory of self‐determination, there is evidence that shows that basic psychological needs play an important role in determining the quality of life of people with HIV.^51^ For example, social support, which indicates the need for a sense of belonging, has a positive relationship with the quality of life of people with HIV. In addition, another study shows that the feeling of competence in managing one's condition and the experience of closeness with one's caregiver are important determinants for the decision to enter care measures while establishing independence in the individual as a component. It is recognized as important for long‐term management in treatment. Also, nurse or caregiver support of HIV‐ positive patients' needs for independence has been shown to predict treatment dependence. Satisfying these three psychological needs leads to sustainable consistent behaviors such as healthy eating and less involvement in incompatible behaviors such as smoking and physical‐mental health including reducing the symptoms of depression, and anxiety and increasing life satisfaction.^52^

The results of the present study showed that the component of mindfulness is a positive and significant predictor of satisfaction of needs and physical health and a negative and significant predictor of sleep quantity. However, about sleep quality and mental health, it does not have predictive power.^16^, ^53^, ^54^ The mindfulness approach has a positive relationship with psychological need satisfaction and also facilitates sleep. Overall, the current results indicate that the only way professionals can help improve health‐related quality of life in HIV patients is through helping to cultivate mindfulness. mind‐awareness helps to accept the signs of satisfying psychological needs and thus provides the possibility of better sleep. Evidence shows that mindfulness with the help of meditation helps to satisfy needs, sleep and improve physical well‐being.^55^ current results suggest that mindfulness may improve health outcomes by facilitating need satisfaction and better sleep quality. Because mindful individuals show greater awareness of current events, they may be more capable of receiving a sense of need satisfaction from events, which in turn predicts better sleep. This finding is consistent with a previous study that examined the relationship between Satisfying basic psychological needs and subjective measures of sleep and daytime dysfunction in adults are consistent. Although no relationship between mindfulness and mental health was found in the present study, the research background shows that mindfulness leads to improvement in the quality and quantity of sleep.^54^

Although lack of mindfulness may predispose people to sleep deprivation, the opposite is also plausible, meaning that sleep deprivation may interfere with people's capacity for mindfulness. The amount of sleep determines consciousness. For example, sleep deprivation leads to reduced attention. Partial sleep deprivation, for example, 5 h of sleep at night, leads to increased distraction in doing work. Considering that mindfulness includes purposeful attention to events and experiences when they occur. These findings indicate that mental awareness is probably disturbed due to sleep deprivation.^56^

Mindfulness has been related to the fulfillment of physical needs. considering the close relationship between physical health and mindfulness, another important aspect of psychological functioning that may affect sleep deprivation is the satisfaction of basic psychological needs. Studies show that high mindfulness leads to the satisfaction of needs, and on the contrary, low mindfulness increases people's sensitivity to the need for failure experiences. Finally, according to the findings, it can be said that higher mindfulness with the help of meditation leads to the improvement of sleep quality and reduces the need for sleep quantity.^57^

### 4.1 Limitations

One of the limitations of this research is that it is a cross‐sectional study, which prevents us from drawing causal conclusions. For example, poor sleep quality may not only undermine physical and mental health but may also be caused by this issue. Future empirical or longitudinal research is needed to address this issue. Overall, the current findings provide preliminary evidence that healthcare professionals seeking to improve individuals' quality of life may focus on helping to develop a more informed approach while supporting basic psychological needs. In this regard, people can be helped to identify and participate in activities that meet daily needs.

## 5 CONCLUSION

HRQOL model can explain 18% of physical health and 16% of mental health in HIV patients in the Iranian Population. The results of the health‐related quality of life model showed that it is possible to help improve mental health and physical health through mindfulness techniques, basic psychological need satisfaction, and improving the quality and quantity of sleep.

## AUTHOR CONTRIBUTIONS

*Study design*: Amir Sam Kianimoghadam. *Collection data*: Seyed Ahmad Alinaghi. *Analysis data*: Amir Sam Kianimoghadam and Abbas Masjedi arani. *Interpretation of data*: Mahsa Abdollahpour and Maryam Bakhtiari. *Writing of the report*: Mahsa Abdollahpour and Seyed Ali Dehghan Manshadi. *The decision to submit the report for publication*: Mohsen Rostami and Minoo Mohraz.

## CONFLICT OF INTEREST STATEMENT

The authors declare no conflict of interest.

## TRANSPARENCY STATEMENT

The lead author Amir Sam Kianimoghadam affirms that this manuscript is an honest, accurate, and transparent account of the study being reported; that no important aspects of the study have been omitted; and that any discrepancies from the study as planned (and, if relevant, registered) have been explained.

## Data Availability

The data that support the findings of this study are available from the corresponding author upon reasonable request.
